# Oncologic Outcomes After Extra-Articular Resection of Bone and Soft Tissue Sarcomas Involving Major Joints: A Systematic Review

**DOI:** 10.3390/cancers18142233

**Published:** 2026-07-12

**Authors:** Carolina Mendez-Guerra, Rayna S. Kuthiala, Andrew R. Moya, Marcos R. Gonzalez, Juan Pretell-Mazzini

**Affiliations:** 1 Facultad de Ciencias de la Salud, Universidad Peruana de Ciencias Aplicadas, Lima 15067, Peru; 2 Dr. Kiran C. Patel College of Osteopathic Medicine, Nova Southeastern University, Davie, FL 33328, USA; rk1131@mynsu.nova.edu; 3 Division of Arthroplasty and Adult Joint Reconstruction, Department of Orthopaedics, University of Miami Miller School of Medicine, Miami, FL 33136, USA; arm577@miami.edu; 4 CEDA Orthopedic Group, Miami, FL 33126, USA; 5 Division of Orthopedic Surgery, Tufts Medical Center, Tufts University School of Medicine, Boston, MA 02111, USA; marcos.gonzalez@tuftsmedicalcenter.org; 6 Division of Orthopedic Oncology, Miami Cancer Institute, Baptist Health System South Florida, Plantation, FL 33324, USA; juan.pretell@baptisthealth.net; 7 Department of Orthopedic Surgery, Herbert Wertheim College of Medicine, Florida International University, Miami, FL 33199, USA

**Keywords:** extra-articular resection, intra-articular sarcoma, bone sarcoma, soft tissue sarcoma, oncologic outcomes

## Abstract

**Background/Objectives**: Extra-articular resection (EAR) may be considered for sarcomas involving or extending into joints, but its oncologic outcomes are not well defined. This study evaluated oncologic outcomes following EAR for bone and soft tissue sarcomas of the knee, shoulder, or hip. **Methods**: This study followed the Preferred Reporting Items for Systematic Reviews and Meta-Analyses guidelines and was registered in the International Prospective Register of Systematic Reviews (ID: CRD420251140969). PubMed and Embase were searched from inception through 14 January 2026, to identify studies including patients with bone or soft tissue sarcomas involving or extending into the knee, shoulder, or hip joints who underwent EAR. Studies including other types of resections or mixed populations were excluded. Primary outcomes were local recurrence (LR) and distant metastasis (DM); secondary outcomes were 1- and 5-year overall survival (OS). Proportions were estimated using sample size-weighted pooling, with 95% confidence intervals (95% CIs) calculated using the Wilson score method. Exploratory subgroup analyses were performed by anatomic location for LR and histologic subtype for DM. Quality assessment was conducted using the Joanna Briggs Institute Critical Appraisal Checklist for case series, and the Newcastle–Ottawa Scale for cohort studies. **Results**: Twenty-one studies, comprising 455 patients, were included. Pooled LR was 11.65% (95% CI, 8.92–15.04), with the highest proportion in the hip (17.65%; 95% CI, 10.53–27.75), followed by the shoulder (10.90%; 95% CI, 6.66–17.13) and knee (9.81%; 95% CI, 6.32–14.80). Pooled DM was 35.26% (95% CI, 30.01–40.87), with the highest proportion in Ewing sarcoma (50.00%; 95% CI, 23.66–76.34) and the lowest in chondrosarcoma (13.79%; 95% CI, 4.51–32.57). No significant differences were found in exploratory subgroup analyses. The 1-year and 5-year OS rates were 92.13% (95% CI, 85.63–95.95) and 58.42% (95% CI, 51.28–65.23), respectively. **Conclusions**: EAR was associated with low reported LR and moderate survival in patients with sarcomas involving or extending into major joints. DM likely reflects underlying tumor biology. These findings should be interpreted cautiously given the lack of a comparison group, heterogeneity of included studies, and statistical limitations.

## Simple Summary

Sarcomas involving or extending into joints may arise from intra-articular structures, extend from adjacent bone and soft tissues, or contaminate the joint. This subset of malignancies is technically demanding, and conventional intra-articular resection may not provide adequate oncologic margins. Extra-articular resection (EAR), involving en bloc removal of the tumor and the entire joint, may be an alternative to amputation in selected patients. Our objective was to review the currently available evidence on local recurrence, distant metastasis, and overall survival after EAR for sarcomas of the knee, shoulder, and hip. We found relatively low local recurrence and moderate survival following this procedure. We also found that the risk of distant spread may be more likely related to underlying tumor biology than to the procedure itself. Despite the limitations of this study, these findings may serve as a benchmark for future studies assessing the role of EAR in this particularly complex population.

## 1. Introduction

Advances in imaging, systemic therapy, and reconstructive techniques have enabled limb salvage surgery (LSS) to largely replace amputation as the standard of care in extremity sarcomas, as it allows for limb preservation while still providing favorable oncologic results [1,2,3,4]. Achieving negative resection margins remains a key principle of sarcoma surgery and is considered essential for optimizing local disease control and overall survival (OS) [5,6,7]. Sarcomas can involve or extend into the joint by originating from intra-articular structures, spreading from surrounding periarticular bone or soft tissue, or by contaminating the joint space secondary to an inappropriately performed procedure or a pathologic fracture [8]. These tumors represent a challenging subset of malignancies for which the optimal surgical approach is not completely defined. In this complex population, conventional intra-articular resection (IAR) may fail to achieve adequate resection margins and may risk tumor seeding when the joint capsule is opened.

Extra-articular resection (EAR) is defined as en bloc resection of the tumor and entire joint without opening the joint capsule [9]. Although technically demanding and often limited to relatively narrow oncologic indications, EAR may be considered for sarcomas involving or extending into the joint when adequate oncologic margins are achievable [10]. The current literature regarding its oncologic outcomes remains limited, as reported outcomes usually come from small and heterogeneous case series and retrospective cohort studies [10,11,12]. Nevertheless, to the best of our knowledge, the proportions of local recurrence (LR), distant metastasis (DM), and OS after EAR, including potential differences by anatomic location or histologic subtype, have not been summarized in the current literature. Therefore, this systematic review sought to synthesize the available evidence on LR, DM, and OS following EAR of bone and soft tissue sarcomas involving or extending into the knee, shoulder, or hip joints, while also exploring potential differences by anatomic location and histologic subtype.

## 2. Materials and Methods

### 2.1. Data Sources and Search Strategy

This systematic review was performed following the Preferred Reporting Items for Systematic Reviews and Meta-Analyses (PRISMA) 2020 guidelines and registered in the International Prospective Register of Systematic Reviews (ID: CRD420251140969), where a short version of the protocol is available. A comprehensive search was conducted in PubMed and Embase from inception through 14 January 2026. The following search terms and Boolean operators were used: ((sarcoma* OR tumor* OR malignan*) AND (joint* OR articula* OR intraarticular OR intra-articular)) AND (“extra-articular resection” OR “extraarticular resection” OR “en bloc resection” OR arthrectom*). The full search strategies for each database are provided in the Supplementary Material (Supplementary Table S1). Citation searching was also performed to identify any potentially missed records.

### 2.2. Eligibility Criteria

For a study to be included, the following criteria needed to be fulfilled: (1) studies including patients with bone or soft tissue sarcomas involving or extending into the knee, shoulder, or hip joints treated with EAR; (2) studies reporting individual or pooled data that could be abstracted; (3) studies having a sample size of at least five patients; and (4) studies published in peer-reviewed journals in English or Spanish.

For studies including mixed populations or resection types, eligible cases were included only when individual-level or subgroup-specific data were available for abstraction. When such data were available, benign lesions, metastatic lesions, and non-EAR procedures were excluded at the patient level rather than excluding the entire study. Nevertheless, an important exception to this rule was applied when a minority of benign or metastatic cases could not be separated from the pooled outcomes and excluding the entire study would have resulted in the loss of a substantial number of otherwise eligible sarcoma patients [13,14,15,16]. Other exclusion criteria included case reports, expert opinions, conference proceedings, review articles, editorials, letters to the editor, unpublished series, and non-peer-reviewed articles.

### 2.3. Study Selection and Data Collection

Two separate search queries were conducted in PubMed and Embase, and the resulting records were uploaded into Covidence (Veritas Health Innovation, Melbourne, Victoria, Australia). Duplicates were removed prior to screening. Two independent reviewers (C.M.-G. and R.S.K.) screened titles and abstracts and then conducted full-text review. References of included studies were also searched to identify any potentially missed records. In cases of disagreement, the senior author (J.P.-M.) was consulted for consensus.

A standardized data extraction form was constructed in Excel, version 16.110.3 (Microsoft Corporation, Redmond, WA, USA). Data were collected independently by two reviewers (C.M.-G. and R.S.K.), and, in cases of discrepancy, the senior author was consulted for consensus. The following variables were extracted: first author, year of publication, sample size, patient age and sex, bone versus soft tissue sarcoma distribution, tumor location and histology, follow-up duration in months, resection margins, LR, DM, and OS at 1, 2, 5, and 10 years. The following variables were quantitatively synthesized: LR, DM, 1-year OS, and 5-year OS. Of note, to quantitatively synthesize DM by histologic type, individual-level data were aggregated.

### 2.4. Primary and Secondary Outcomes

The primary outcomes were LR and DM, and the secondary outcomes were OS at the 1-year, 2-year, 5-year, and 10-year time points. The events of LR and DM were recorded as reported in the included studies. The proportions of LR and DM were calculated as the number of events divided by the number of patients included in each study; if more than one event occurred per patient, only the first event was counted. OS was recorded as reported in the included studies [11,12,15,17,18] or was extracted from Kaplan–Meier curves [10,16,19,20]. Importantly, OS values should be interpreted as time-to-event estimates based on patients remaining at risk at each time point, rather than as evidence that all patients completed follow-up for that specific time frame.

### 2.5. Study Identification and Characterization

**Figure 1:**
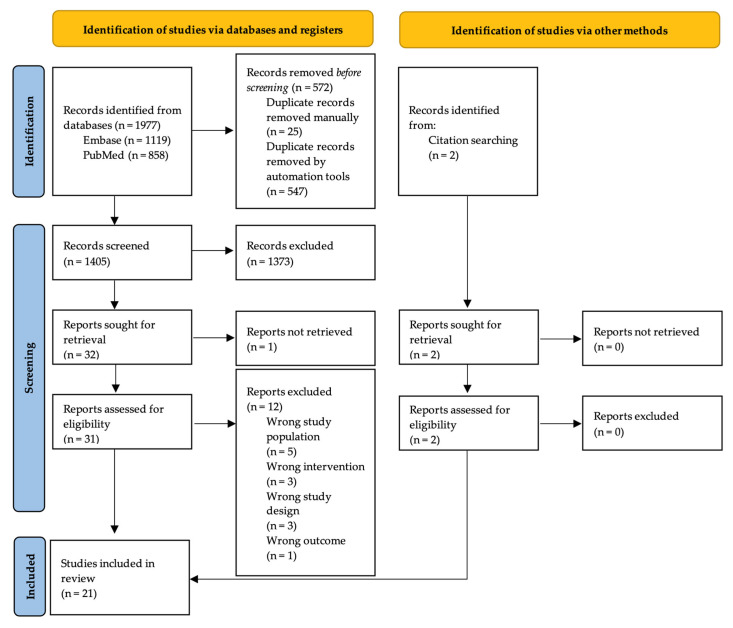
PRISMA Flow Diagram of Study Selection. PRISMA: Preferred Reporting Items for Systematic Reviews and Meta-Analyses.

The initial database search yielded 1977 records: 1119 from Embase and 858 from PubMed. After deduplication (*n* = 572), 1405 records were screened, resulting in the exclusion of 1373. A total of 32 reports were sought for retrieval; one report could not be retrieved. Full-text review was conducted on 31 reports. Twelve reports were excluded after full-text review: 5 because of wrong study population [21,22,23,24,25], 3 because of wrong intervention [26,27,28], 3 because of wrong study design [29,30,31], and 1 because of wrong outcome [32]. Additionally, two records were identified through citation searching and were ultimately included. A total of 455 patients from 21 studies were included in this systematic review (Figure 1) [8,9,10,11,12,13,14,15,16,17,18,19,20,33,34,35,36,37,38,39,40].

The earliest study was published in 1990 [13], while the most recent was published in 2026 [40]. The sample size of included studies ranged from 6 [19] to 59 [14]. The patient age ranged from 17.9 [11] to 54.0 [15], with the majority of studies reporting an average age in the third to fifth decades of life [8,9,10,12,13,14,15,16,17,18,19,20,35,36,38,39,40]; ages seemed to vary by anatomic location, with consistently younger ages in the studies including patients with sarcomas of the shoulder, compared with the knee and hip joints. Most studies included a predominance of bone sarcomas, with proportions exceeding 50% in the majority of cohorts [8,10,11,12,13,15,17,19,20,33,34,35,36,37,39,40]. The majority of studies reported tumors arising from the osseous components of the joint and extending into it; tumor origin from the surrounding soft tissue [8,16,17,38] or intra-articular location was reported to a much lesser extent [14,16,18,35]. Follow-up duration ranged from 22.5 [13] to 101.0 months (Table 1) [16].

Osteosarcoma was the most frequently reported histology across included studies, accounting for 213 (47%) cases, followed by chondrosarcoma with 116 (25%) cases. Other less frequently represented histologies were undifferentiated pleomorphic sarcoma with 30 (7%) cases, synovial sarcoma with 25 (5%) cases, and Ewing sarcoma with 19 (4%) cases. The remaining 52 (11%) cases comprised a heterogeneous group of histologies, including angiosarcoma, clear cell sarcoma, chondroblastoma, epithelioid hemangioendothelioma, epithelioid sarcoma, fibrocartilaginous hamartoma, fibrosarcoma, giant cell tumor, hemangiopericytoma, leiomyosarcoma, liposarcoma, malignant peripheral nerve sheath tumor, metastatic carcinoma, myxofibrosarcoma, pigmented villonodular synovitis, rhabdomyosarcoma, and spindle cell/undifferentiated sarcoma (Table 2).

Reconstruction strategies and soft-tissue reinforcement techniques varied across anatomic locations. In the knee, all included studies reported endoprosthetic reconstruction, using either distal femoral or proximal tibial replacement depending on tumor location. Cemented wedges used to reconstruct proximal tibial defects were reported in 2 studies [14,18], whereas the use of an allograft–prosthetic composite incorporating the entire extensor mechanism (EM) was reported in 1 study [38]. Interestingly, a novel surgical technique involving coronal splitting of the patella to preserve the EM while maintaining adequate EAR margins was described in 2 studies [9,16]. The use of a gastrocnemius flap as a soft-tissue reinforcement technique was reported in all 10 studies; numerical data were available in 8 studies [14,16,17,18,19,34,36,38], comprising a total of 87 patients (41%). Around the shoulder, proximal humeral replacement was the most frequently reported reconstruction strategy, described in 7 studies [11,13,20,33,35,37,39]. The Trevira tube was the most commonly reported soft-tissue reinforcement technique, described in 3 studies [20,35,40]. Around the hip, reconstruction generally consisted of proximal or total femoral replacement combined with acetabular or pelvic reconstruction. Regarding neoadjuvant or adjuvant therapy, the reported use of radiotherapy ranged from 0% [34,36,38] to 45% [9], while chemotherapy ranged from 18% [15] to 100% across the included studies [17,19,33,37] (Table 3).

Resection margin status was reported in 17 studies [8,10,11,12,13,14,15,16,17,18,19,20,33,35,38,39,40], of which 15 used wide, marginal, and intralesional classifications. Among studies using this classification, 7 reported wide margins in all included patients [14,16,17,18,19,33,35]. Marginal and intralesional resections were reported in 8 [8,10,11,13,15,20,38,39] and 4 studies [8,10,15,20], respectively. By anatomic location, marginal resections were reported in 21 knee cases (12.5%), 18 shoulder cases (15.3%), and 18 hip cases (34.6%), whereas intralesional resections were reported in 3 knee cases (1.8%), 3 shoulder cases (2.5%), and 4 hip cases (7.7%) (Table 4).

### 2.6. Statistical Analysis

Sample size-weighted pooled proportions for LR, DM, 1-year OS, and 5-year OS were estimated, with 95% confidence intervals (95% CIs) calculated using the Wilson score method with continuity correction. Exploratory subgroup analyses by joint for LR and histologic subtype for DM were conducted. Differences between these groups were explored using the chi-square test or Fisher’s exact test, based on calculated expected values. A sensitivity analysis excluding studies reporting a minority of benign or metastatic cases was performed to assess the robustness of the pooled proportions of LR and DM. OS at 1 and 5 years was not included in the sensitivity analysis, as the primary analysis did not include studies reporting benign or metastatic cases. A *p*-value < 0.05 was considered statistically significant. All statistical analyses were performed in R, version 4.6.0 (R Foundation for Statistical Computing, Vienna, Austria).

### 2.7. Quality Assessment

Quality assessment was conducted using the Joanna Briggs Institute (JBI) Critical Appraisal Checklist for case series, and the Newcastle–Ottawa Scale (NOS) for cohort studies [41,42]. The JBI Critical Appraisal Checklist comprises 10 questions, with an overall appraisal determined by the reviewer. The NOS comprises three domains and has a maximum attainable score of 9 points, with scores ≥7 considered high quality. Two independent reviewers (C.M.-G. and A.R.M.) conducted the quality assessment, and, in cases of discrepancy, the senior author (J.P.-M.) was consulted. No articles were excluded based on quality assessment (Supplementary Tables S2 and S3).

## 3. Results

### 3.1. What Is the Proportion of LR Among Patients with Bone and Soft Tissue Sarcomas Involving or Extending into the Knee, Shoulder, or Hip Joints Following EAR, and Does It Vary by Anatomic Location?

The proportion of LR ranged from 0% [17,18,19,34,37,40] to 29% [10] across included studies (Table 4). The pooled proportion of LR was 11.65% (95% CI, 8.92–15.04) (Table 5). By anatomic location, the highest proportion was estimated in the hip (17.65%, 95% CI, 10.53–27.75), followed by the shoulder (10.90%, 95% CI, 6.66–17.13) and the knee (9.81%, 95% CI, 6.32–14.80). No statistically significant differences were found by anatomic location (*p*-value = 0.153) (Table 6). Given the small sample sizes and lack of adjustment for study-level clustering, this subgroup analysis should be considered exploratory and likely underpowered to detect true differences between groups.

In the sensitivity analysis excluding studies reporting a minority of benign or metastatic cases, the pooled proportion of LR was 13.31% (95% CI, 9.83–17.74). By anatomic location, LR was 13.60% for the knee (95% CI, 8.35–21.16), 12.12% for the shoulder (95% CI, 7.30–19.22), and 15.69% for the hip (95% CI, 7.48–29.14) (Table 7). No statistically significant differences were found by anatomic location (*p*-value = 0.811). The sensitivity analysis yielded findings consistent with the primary analysis.

### 3.2. What Is the Proportion of DM Among Patients with Bone and Soft Tissue Sarcomas Involving or Extending into the Knee, Shoulder, or Hip Joints Following EAR, and Does It Vary by Histologic Subtype?

The proportion of DM ranged from 9% [40] to 56% [20] across included studies (Table 4). The pooled proportion of DM was 35.26% (95% CI, 30.01–40.87) (Table 5). By histologic subtype, the highest proportion was estimated in Ewing sarcoma (50.00%, 95% CI, 23.66–76.34), followed by soft tissue sarcomas (35.29%, 95% CI, 22.80–50.00), osteosarcoma (30.77%, 95% CI, 21.08–42.38), and chondrosarcoma (13.79%, 95% CI, 4.51–32.57). No statistically significant differences were found by histologic subtype (*p*-value = 0.088) (Table 6). This analysis should be interpreted as exploratory and likely underpowered due to reduced sample sizes and lack of adjustment for study-level clustering.

In the sensitivity analysis excluding studies reporting a minority of benign or metastatic cases, the pooled proportion of DM was consistent with the primary analysis (36.05%, 95% CI, 30.25–42.27) (Table 7).

### 3.3. What Is the OS of Patients with Bone and Soft Tissue Sarcomas Extending into the Knee, Shoulder, or Hip Joints Following EAR?

The 1-year OS ranged from 78% [16] to 100% [10,17,18,19], and the 5-year OS ranged from 42% [20] to 100% [18] (Table 4). The pooled proportion of 1-year OS was 92.13% (95% CI, 85.63–95.95), while the pooled proportion of 5-year OS was 58.42% (95% CI, 51.28–65.23) (Table 5).

## 4. Discussion

Sarcomas involving or extending into the joint constitute a particularly challenging subset of malignancies for which the optimal surgical management is not yet completely elucidated. By originating from critical intra- or peri-articular structures or secondary-to-joint-space contamination, these tumors make conventional IAR potentially inadequate for achieving wide oncologic margins. In this setting, EAR may represent a limb-sparing surgical technique in which the entire tumor and joint are removed en bloc, without opening the joint capsule. The surgical principle that wide negative margins are essential for achieving local control constitutes the conceptual foundation for EAR in this setting. However, despite this rationale, the oncologic outcomes of EAR in this specific population remain incompletely summarized. Therefore, this systematic review evaluated oncologic outcomes after EAR for sarcomas of the knee, shoulder, and hip. We found pooled proportions of 12% for LR and 35% for DM, with pooled 1-year and 5-year OS of 92% and 58%, respectively. Exploratory subgroup analyses by anatomic location for LR and histologic subtype for DM showed no statistically significant differences; however, these analyses should be interpreted as hypothesis-generating and likely underpowered due to small sample sizes and lack of adjustment for study-level clustering.

Direct comparisons between EAR and primary amputation for sarcomas involving or extending into the joint remain inadequately studied. Therefore, amputation series for extremity bone and soft tissue sarcomas are used as contextual references in the following paragraphs. Importantly, patients undergoing amputation often represent a distinct oncologic population, including cases with more aggressive disease, neurovascular involvement, or failed prior LSS; thus, this contextualization should not be interpreted as a direct comparison.

The pooled proportion of LR was 12%. This finding suggests that EAR may be associated with relatively low reported local recurrence in selected patients, although its interpretation remains limited by the observational nature and heterogeneity of the included studies. Moreover, the observed LR proportion falls within the range of previously reported outcomes after amputation for extremity sarcomas; however, no direct comparison can be made because amputation cohorts represent a distinct oncologic population [43,44]. Exploratory subgroup analysis showed numerical variation in LR by anatomic location, with the highest proportion observed in the hip. The three-dimensional anatomy of the pelvis, coupled with its proximity to major neurovascular bundles and pelvic viscera, challenges the achievement of clear margins in pelvic tumor resections, especially in tumors compromising the periacetabular zone, which could in turn hinder local disease control [45]. In line with this, although the association between margin status and LR could not be assessed in this review, marginal and intralesional resections were descriptively reported more often following EAR of the hip than of the knee and shoulder joints. In addition, joint effraction following EAR for proximal femur tumors was reported more commonly, which may be relevant to the higher observed proportion of LR in the hip [12].

LR following EAR of the knee joint was generally reported between 0% and 10% [9,14,16,17,18,19,34,36], with only Capanna et al. and Shahid et al. reporting higher proportions of 21% and 29%, respectively [10,38]. This may be partially explained by the fact that EAR of the knee joint benefits from a more standardized procedure, in which the joint is resected en bloc with either complete patellectomy or coronal splitting of the patella. In one study comparing IAR, EAR, and amputation for sarcomas of the knee joint, no statistically significant differences were found in oncologic outcomes; however, local recurrence-free survival was 69% in the EAR group, 86% in the IAR group, and 100% in the amputation group [10]. This numerical difference may be partially explained by margin status, as marginal and intralesional margins were more frequently reported in the EAR group [10]. Collectively, the exploratory variation observed in LR across anatomic locations may reflect differences in anatomic complexity and margin feasibility, rather than the superiority of a specific surgical approach for a given location.

The pooled proportion of DM was 35%. Prior amputation series have reported DM rates of approximately 54% in extremity sarcomas [43]; however, this should be interpreted only as a contextual reference because amputation cohorts often include more complex patients. Unlike LR, which may correlate more closely with the oncologic adequacy of the surgical procedure, DM likely reflects underlying tumor biology rather than a procedure-specific outcome. In this setting, histologic subtype and grade, as well as tumor size and depth, are among the strongest predictors for metastatic dissemination [46,47]. Moreover, DM by histologic subtype was estimated using aggregated individual-level data. The highest proportion of DM was observed in Ewing sarcoma, and the lowest was observed in chondrosarcoma. This pattern is consistent with the literature, which describes Ewing sarcoma as an aggressive malignancy with high metastatic potential, whereas low-grade chondrosarcomas often behave as slow-growing tumors that metastasize less frequently [48,49,50,51]. Importantly, our subgroup analysis should be interpreted as exploratory, as it did not account for study-level clustering and was likely underpowered due to reduced sample sizes.

The pooled 1-year and 5-year OS was 92% and 58%, respectively. This finding suggests that EAR may be associated with moderate survival outcomes in this selected population. Although no direct comparison between EAR and amputation could be performed, evidence in extremity bone and soft tissue sarcomas has reported comparable or improved survival after LSS compared with amputation. Prior meta-analyses have shown nearly twice the odds of 5-year OS following LSS compared with amputation in osteosarcoma [2]. Similarly, LSS significantly improved both OS and cancer-specific survival in patients with limb osteosarcoma compared with amputation [3]. In extremity soft tissue sarcoma, no disease-specific survival benefit has been found in patients who underwent amputation, supporting the role of LSS as an alternative for selected cases [4]. Collectively, although these findings cannot be directly extrapolated to sarcomas involving or extending into the joint, they support the need for future comparative studies evaluating EAR versus amputation in this specific population to better define the oncologic role of EAR while minimizing selection bias.

To provide an additional contextual framework for the interpretation of our findings, we summarized oncologic outcomes, including LR, DM, and 5-year OS, from included studies reporting both EAR and IAR cohorts. It is important to highlight that these data should be interpreted as a contextual reference rather than as a comparative, since patients undergoing IAR as LSS represent a distinct surgical subgroup in whom tumor extent, joint involvement, and margin feasibility may differ from those requiring EAR. In the knee, LR ranged from 0% [19,34,36] to 19% [10] in the IAR group, whereas it ranged from 0% [19,34] to 29% [10] in the EAR group. DM ranged from 0% [36] to 43% [10] in the IAR group and from 7% [34] to 50% [19] in the EAR group. Among studies reporting 5-year OS, it ranged from 67% [10] to 85% [19] in the IAR group and from 60% [10] to 80% [19] in the EAR group. In the shoulder, LR ranged from 0% [11] to 10% [33] in the IAR group, whereas it ranged from 10% [33] to 12% [11] in the EAR group. DM was reported by only one study, with a value of 50% in the IAR group and 10% in the EAR group [33]. Five-year OS was also reported by only one study, with a value of 77% in the IAR group and 59% in the EAR group [11]. In the hip, LR was reported by only one study, with a value of 37% in the IAR group and 29% in the EAR group [52]. Taken together, LR and DM showed overlapping ranges between IAR and EAR cohorts across anatomic locations, whereas 5-year OS was numerically lower in the EAR group among studies reporting this outcome. Importantly, this finding should not be interpreted as evidence that EAR worsens survival, as patients requiring this procedure typically represent a more complex subset. This contextual framework highlights the need for future comparative studies with adequately matched cohorts to support more definitive conclusions (Table 8).

The lack of a comparison group is an important limitation. Amputation performed specifically for sarcomas involving or extending into the joint represents the ideal comparison group; however, the current amputation literature includes a heterogeneous population, precluding its use as a comparison group. Moreover, even if such a comparison group were available, selection bias would remain an important concern, as patients selected for EAR may represent a more favorable surgical subgroup a priori compared with patients requiring amputation. This could lead to overestimation of the favorable oncologic outcomes associated with EAR. The observational and retrospective nature of the included studies is also a limitation, as no causal effect can be established based on our results. The heterogeneity of the included study population must also be disclosed, as studies varied substantially in histologic subtype and grade, anatomic location, follow-up, and sample size. This review encompasses different tumor histologies, including osteosarcoma, chondrosarcoma, and undifferentiated pleomorphic sarcoma, among others, each characterized by distinct biological behavior, metastatic potential, response to systemic and local therapy, and prognosis. In addition, we included studies reporting a minority of benign and metastatic cases treated with EAR of the joint when individual-level or subgroup-specific data were not available and excluding the entire study would have resulted in the loss of a substantial number of eligible sarcoma patients. Although this approach preserved the sample size of the analysis, it further increased the clinical heterogeneity of the included population. To mitigate the impact of this limitation, the following measures were taken. First, we performed a sensitivity analysis excluding studies that included benign or metastatic cases to assess the robustness of our pooled estimates for overall LR, LR by anatomic location, and DM. The results of this sensitivity analysis were consistent with our primary analysis. Second, for DM, we extracted individual-level data whenever available to estimate metastatic risk according to histologic subtype. Although these analyses were exploratory and likely underpowered, they provide a more clinically meaningful description of metastatic risk by histologic subtype following EAR. Accordingly, the overall estimates should be interpreted cautiously as descriptive estimates across a highly heterogeneous population; in particular, the overall DM estimate should not be considered a histology-specific estimate.

Another important limitation that should be disclosed is the inconsistent reporting of follow-up across included studies. Most studies reported only the mean or median follow-up duration without specifying its starting point. Besides introducing further clinical heterogeneity, this also prevented standardization of follow-up definitions across the included studies. In addition, the assessment of LR, DM, and OS according to resection margin status was not feasible in our systematic review, and this should be disclosed as an important limitation. Individual-level data with complete information on oncologic outcomes and resection margin status were available only for a small subset of patients, which, coupled with the extremely low number of marginal and intralesional resections, prevented meaningful assessment of this association.

Statistical limitations should also be acknowledged. Our pooled estimates were calculated as descriptive sample size-weighted pooled proportions rather than as formal meta-analytic estimates. Although we initially attempted to perform a random-effects meta-analysis of proportions, the models demonstrated instability in the estimation of between-study variance, I^2^, and pooled 95% CIs, likely due to small sample sizes, sparse event counts, and multiple zero- or boundary-event proportions. Importantly, because these estimates were derived from descriptive pooling rather than a formal meta-analytic model, they should be interpreted as descriptive summary measures that do not account for between-study heterogeneity. In addition, subgroup comparisons should be considered exploratory given the limited statistical power and lack of adjustment for study-level clustering. Importantly, although the Wilson score method was used to calculate the pooled 95% CIs, which is appropriate in the setting of small samples and zero- or boundary-event proportions, this approach neither accounts for study-level clustering nor overcomes the limited power of the subgroup analyses. Finally, publication bias cannot be excluded, as small retrospective surgical series with negative, inconclusive, or unfavorable outcomes may be underreported in the published literature. Ideally, formal publication bias assessment should be performed when conducting a meta-analysis; however, our study design, coupled with small sample sizes, zero- or boundary-event proportions, and clinical heterogeneity for certain outcomes, could have resulted in unreliable or misleading findings if such an assessment had been conducted.

## 5. Conclusions

EAR for sarcomas involving or extending into the knee, shoulder, or hip joints was associated with relatively low reported LR and moderate survival outcomes. DM likely reflects underlying tumor biology rather than a procedure-specific outcome. These findings should be interpreted cautiously given the lack of a comparison group, heterogeneity of the included studies, and inherent statistical limitations. Future comparative studies evaluating EAR and amputation in this specific population are needed to better define the oncologic role of EAR.

## Figures and Tables

**Table 1:** Baseline characteristics of included studies, stratified by anatomic location.

First Author	Year	LOE	Sample (*n*)	Age *	Sex (M:F)	Bone Sarcoma, *n* (%)	Tumor Location (%)	Follow-Up (Month) *
Knee (*n* = 214)								
Kawai et al. [34]	1998	IV	28	26.8	16:12	24 (86)	DF (100)	97.3
Kendall et al. [36]	2000	III	9	32.7	6:3	7 (78)	DF (56), PT (44)	30.0
Anract et al. [17]	2001	IV	9	36.0	6:3	5 (56)	DF (67), PT (22), ST (11)	23.0
Capanna et al. [38]	2011	IV	12	37.3	6:6	5 (42)	ST (58), DF (17), PT (17), Patella (8)	40.1
Zwolak et al. [9]	2011	IV	11	39.8	6:5	5 (45)	DF (100)	37.5
Hardes et al. [14]	2013	IV	59	33.0	36:23	51 (86) ^†^	DF (83), Intra-articular (7), PT (5), ST (3), Patella (2)	56.4
Ieguchi et al. [19]	2014	IV	6	44.4	5:1	6 (100)	PF (83), PT (17)	82.8
Shahid et al. [10]	2017	III	42	33.0	22:20	35 (83)	DF, PT (not specified)	64.0 ^‡^
Nottrott et al. [18]	2019	IV	8	47.0 ^§^	4:4	0 (0)	Intra-articular (100)	80.0 ^§^
Zoccali et al. [16]	2026	III	30	40.0	13:17	19 (63) ^†^	DF (67), PT (17), Intra-articular (10), Patella (3), ST (3)	101.0 ^§^
Shoulder (*n* = 156)								
Capanna et al. [13]	1990	IV	24	34.3	13:11	22 (92) ^†^	PH (100)	22.5
Meller et al. [33]	1997	IV	10	18.4	6:4	10 (100)	PH (100)	37.6
Voggenreiter et al. [35]	1999	IV	17	40.8	13:4	11 (65)	PH (48), Scapula (42), Clavicle (5), ACJ (5)	55.4
Wittig et al. [37]	2002	IV	22	21.7	11:11	22 (100)	PH (100)	92.0
Xie et al. [39]	2014	IV	23	34.7	14:9	14 (61)	NR	40.3
Böhler et al. [11]	2018	III	17	17.9 ^‡,§^	27:22 ^‡^	17 (100)	PH (100)	63.8 ^‡,§^
Tsuda et al. [20]	2020	III	32	39.0 ^§^	17:15	32 (100)	PH (69), Scapula (31)	60.0 ^§^
Samargandi et al. [40]	2025	IV	11	47.5	4:7	8 (73)	PH (82), Scapula (18)	41.9
Hip (*n* = 85)								
Li et al. [8]	2018	IV	18	39.6	13:5	14 (78)	PF (50), Pelvis (44), ST (6)	35.1 ^¶^
Fujiwara et al. [15]	2020	III	34	54.0 ^§^	27:7	32 (94) ^†^	Pelvis (76), PF (24)	38.0 ^§^
Housset et al. [12]	2022	III	33	52.0 ^§^	19:14	33 (100)	Pelvis (58), PF (42)	76.0 ^§^

ACJ: acromioclavicular joint; DF: distal femur; LOE: level of evidence; NR: not reported; PF: proximal femur; PH: proximal humerus; PT: proximal tibia; ST: soft tissue. * Values were reported as means unless otherwise specified. ^†^ The remaining cases do not exclusively correspond to soft tissue sarcomas and include a minority of other conditions; see Table 2 for detailed histologic distribution. ^‡^ Values were reported for a larger study cohort and not limited to the population of interest. ^§^ Values were reported as medians. ^¶^ Values were reported based on 17 patients due to one perioperative death.

**Table 2:** Histologic distribution of tumors across included studies, stratified by anatomic location.

First Author	Sample (*n*)	OSA, *n* (%)	CS, *n* (%)	UPS, *n* (%)	SS, *n* (%)	ES, *n* (%)	Other, *n* (%) *
Knee (*n* = 214)							
Kawai et al. [34]	28	20 (71)	3 (11)	4 (14)	0 (0)	1 (4)	0 (0)
Kendall et al. [36]	9	4 (45)	2 (22)	1 (11)	1 (11)	0 (0)	1 (11)
Anract et al. [17]	9	5 (56)	0 (0)	0 (0)	0 (0)	0 (0)	4 (44)
Capanna et al. [38]	12	3 (25)	0 (0)	1 (8)	4 (33)	2 (17)	2 (17)
Zwolak et al. [9]	11	4 (37)	1 (9)	2 (18)	1 (9)	0 (0)	3 (27)
Hardes et al. [14] ^†^	59	34 (58)	7 (12)	7 (12)	7 (12)	0 (0)	4 (6)
Ieguchi et al. [19]	6	6 (100)	0 (0)	0 (0)	0 (0)	0 (0)	0 (0)
Shahid et al. [10]	42	25 (60)	8 (19)	0 (0)	0 (0)	2 (5)	7 (16)
Nottrott et al. [18]	8	0 (0)	0 (0)	1 (13)	5 (63)	0 (0)	2 (24)
Zoccali et al. [16] ^†^	30	13 (43)	3 (10)	0 (0)	3 (10)	2 (7)	9 (30)
Shoulder (*n* = 156)							
Capanna et al. [13] ^†^	24	9 (38)	8 (33)	3 (13)	0 (0)	0 (0)	4 (16)
Meller et al. [33]	10	9 (90)	0 (0)	0 (0)	0 (0)	1 (10)	0 (0)
Voggenreiter et al. [35]	17	1 (6)	7 (41)	3 (18)	1 (6)	3 (18)	2 (11)
Wittig et al. [37]	22	22 (100)	0 (0)	0 (0)	0 (0)	0 (0)	0 (0)
Xie et al. [39]	23	4 (17)	7 (31)	1 (4)	3 (13)	3 (13)	5 (22)
Böhler et al. [11]	17	17 (100)	0 (0)	0 (0)	0 (0)	0 (0)	0 (0)
Tsuda et al. [20]	32	14 (44)	13 (41)	0 (0)	0 (0)	3 (9)	2 (6)
Samargandi et al. [40]	11	3 (27)	5 (46)	2 (18)	0 (0)	0 (0)	1 (9)
Hip (*n* = 85)							
Li et al. [8]	18	7 (39)	7 (39)	3 (17)	0 (0)	0 (0)	1 (5)
Fujiwara et al. [15] ^†^	34	3 (9)	25 (73)	2 (6)	0 (0)	1 (3)	3 (9)
Housset et al. [12]	33	10 (30)	20 (61)	0 (0)	0 (0)	1 (3)	2 (6)

CS: chondrosarcoma; ES: Ewing sarcoma; OSA: osteosarcoma; SS: synovial sarcoma; UPS: undifferentiated pleomorphic sarcoma. * Other histologies included angiosarcoma, clear cell sarcoma, chondroblastoma, epithelioid hemangioendothelioma, epithelioid sarcoma, fibrocartilaginous hamartoma, fibrosarcoma, giant cell tumor, hemangiopericytoma, leiomyosarcoma, liposarcoma, malignant peripheral nerve sheath tumor, metastatic carcinoma, myxofibrosarcoma, pigmented villonodular synovitis, rhabdomyosarcoma, and spindle cell/undifferentiated sarcoma. ^†^ Hardes et al. [14] included 1 case of giant cell tumor; Zoccali et al. [16] included 3 cases of solitary metastases and 2 cases of giant cell tumor; Capanna et al. [13] included 1 case of Wilms metastasis and 1 case of fibrocartilaginous hamartoma; and Fujiwara et al. [15] included 1 case of metastatic renal cell carcinoma; these cases were ultimately included in the analysis.

**Table 3:** Reconstruction strategies, soft-tissue reinforcement, and neoadjuvant and adjuvant therapy, stratified by anatomic location.

First Author	Sample, *n* (%)	Reconstruction Strategy	Soft-Tissue Reinforcement, *n* (%)	RT, *n* (%) ^†^	CT, *n* (%) ^†^
Knee (*n* = 214)					
Kawai et al. [34]	28	DFR	Sartorius flap, 7 (25); gastrocnemius flap, 6 (21); latissimus dorsi flap, 2 (7)	0 (0)	24 (86)
Kendall et al. [36]	9	DFR/PTR	Gastrocnemius flap, 3 (33); semitendinosus transfer, 3 (33); semimembranosus transfer, 1 (11)	0 (0)	NR
Anract et al. [17]	9	DFR/PTR	Gastrocnemius flap, 9 (100); pes anserinus transfer, 4 (44)	NR	9 (100)
Capanna et al. [38]	12	DFR + PT APC with whole EM	Gastrocnemius flap, 1 (8); anterolateral thigh flap, 2 (17)	0 (0)	9 (75)
Zwolak et al. [9]	11	DFR with native EM preservation	Gastrocnemius flap, *n* (%) NR	5 (45)	7 (64)
Hardes et al. [14]	59	DFR with cemented wedges for PT defects/PTR	Gastrocnemius flap, 53 (90); Trevira tube, 10 (17)	3 (5)	42 (71)
Ieguchi et al. [19]	6	DFR/PTR	Gastrocnemius flap, 1 (17)	1 (17)	6 (100)
Shahid et al. [10]	42	DFR/PTR	Gastrocnemius flap, *n* (%) NR	NR	NR
Nottrott et al. [18]	8	DFR with cemented wedges for PT defects/PTR	Gastrocnemius flap, 7 (88); Trevira tube, 4 (50); mesh graft, 3 (38)	3 (38)	4 (50)
Zoccali et al. [16]	30	EM excision group: arthrodesis prosthesis or DFR/PTR Patellar coronal osteotomy group: DFR/PTR	Gastrocnemius flap, 7 (23); PT allograft, 1 (3); fascia lata allograft, 6 (20)	2 (7)	20 (67)
Shoulder (*n* = 156)					
Capanna et al. [13]	24	Classical T-L: PHR or intramedullary rod–cement reconstruction Modified T-L: PHR	NR	1 (4)	12 (50)
Meller et al. [33]	10	PHR/improvised implant/autograft reconstruction	NR	1 (10)	10 (100)
Voggenreiter et al. [35]	17	Classical/modified T-L: PHR	Trevira tube, *n* (%) NR	3 (18)	5 (29)
Wittig et al. [37]	22	PHR with static/dynamic suspension	NR	NR	22 (100)
Xie et al. [39]	23	PHR with simple suspension technique	LARS, 9 (27) *	NR	16 (70)
Böhler et al. [11]	17	PHR	LARS, 14 (29); fascia lata autograft, 7 (14); Vicryl mesh, 3 (6) *	NR	46 (94) *
Tsuda et al. [20]	32	PHR/excision arthroplasty	Trevira tube, *n* (%) NR; Mersilene mesh, *n* (%) NR	NR	14 (44)
Samargandi et al. [40]	11	Cement spacer humeral suspension	Trevira tube, 11 (100)	2 (18)	3 (27)
Hip (*n* = 85)					
Li et al. [8]	18	Hemipelvic EP + PFR/TFR	NR	NR	7 (39)
Fujiwara et al. [15]	34	Acetabular reconstruction + PFR/conventional femoral stem	NR	5 (15)	6 (18)
Housset et al. [12]	33	PFR/APC/standard stem + acetabular/pelvic reconstruction	NR	2 (6)	10 (30)

APC, allograft–prosthetic composite; CT, chemotherapy; DFR, distal femoral replacement; EM, extensor mechanism; EP, endoprosthesis; LARS, Ligament Advanced Reinforcement System; NR, not reported; PFR, proximal femoral replacement; PHR, proximal humeral replacement; PT, proximal tibia; PTR, proximal tibial replacement; RT, radiotherapy; TFR, total femoral replacement; T-L, Tikhoff–Linberg procedure. * Values were reported for a larger study cohort and not limited to the population of interest. ^†^ RT and CT include both preoperative and postoperative treatment.

**Table 4:** Study-level oncologic outcomes following extra-articular resection of sarcomas involving the knee, shoulder, or hip joints.

First Author	Sample (*n*)	Resection Margins, *n* (%) *	Local Recurrence, *n* (%)	Metastasis, *n* (%)	1-Year OS (%)	2-Year OS (%)	5-Year OS (%)	10-Year OS (%)
Knee (*n* = 214)								
Kawai et al. [34]	28	NR	0 (0)	2 (7)	NR	NR	NR	NR
Kendall et al. [36]	9	NR	1 (11)	3 (33)	NR	NR	NR	NR
Anract et al. [17]	9	W, 9 (100)	0 (0)	2 (22)	100	100	NR	NR
Capanna et al. [38]	12	W, 11 (92); M, 1 (8)	3 (25)	4 (33)	NR	NR	NR	NR
Zwolak et al. [9]	11	NR	1 (9)	6 (55)	NR	NR	NR	NR
Hardes et al. [14]	59	W, 59 (100)	2 (3)	NR	NR	NR	NR	NR
Ieguchi et al. [19]	6	W, 6 (100)	0 (0)	3 (50)	100	100	80	80
Shahid et al. [10]	42	W, 19 (45); M, 20 (48); I, 3 (7)	12 (29)	18 (43)	100	93	60	46
Nottrott et al. [18]	8	W, 8 (100)	0 (0)	2 (25)	100	100	100	NR
Zoccali et al. [16]	30	W, 30 (100)	2 (7)	12 (40)	78	68	48	48
Shoulder (*n* = 156)								
Capanna et al. [13]	24	W, 22 (92); M, 2 (8)	1 (4)	5 (21)	NR	NR	NR	NR
Meller et al. [33]	10	W, 10 (100)	1 (10)	1 (10)	NR	NR	NR	NR
Voggenreiter et al. [35]	17	W, 17 (100)	2 (12)	8 (47)	NR	NR	NR	NR
Wittig et al. [37]	22	NR	0 (0)	10 (45)	NR	NR	NR	NR
Xie et al. [39]	23	W, 17 (74); M, 6 (26)	5 (22)	8 (35)	NR	NR	NR	NR
Böhler et al. [11]	17	W, 15 (88); M, 2 (12)	2 (12)	NR	NR	NR	59	59
Tsuda et al. [20]	32	W, 16 (50); M, 8 (25); I, 3 (9) ^†^	6 (19)	18 (56)	92	78	42	42
Samargandi et al. [40]	11	R0, 10 (91); R1, 1 (9)	0 (0)	1 (9)	NR	NR	NR	NR
Hip (*n* = 85)								
Li et al. [8]	18	W, 13 (72); M, 4 (22); I, 1 (6)	4 (22)	7 (39)	NR	NR	NR	NR
Fujiwara et al. [15]	34	W, 17 (50); M, 14 (41); I, 3 (9)	7 (21)	NR	NR	NR	54	NR
Housset et al. [12]	33	R0, 30 (91); non-R0, 3 (9)	4 (12)	NR	NR	NR	76	NR

I: intralesional; M: marginal; non-R0: margin reported as not microscopically negative; OS: overall survival; R0: microscopically negative margin; R1: microscopically positive margin; W: wide. * Samargandi et al. [40] and Housset et al. [12] reported surgical margins using the residual tumor (R) classification. ^†^ Surgical margins were not available in 5 patients.

**Table 5:** Sample Size-Weighted Pooled Oncologic Outcomes Following Extra-Articular Resection of Knee, Shoulder, and Hip Sarcomas.

Outcome	Events/Total (n/N)	Estimate (%) *	95% CI ^†^
Local recurrence	53/455	11.65	8.92–15.04
Distant metastasis	110/312	35.26	30.01–40.87
1-year overall survival	117/127	92.13	85.63–95.95
5-year overall survival	118/202	58.42	51.28–65.23

CI: Confidence interval. * Estimates were calculated using sample size-weighted pooled proportions. ^†^ Confidence intervals were calculated using the Wilson score method with continuity correction.

**Table 6:** Subgroup Analysis of Local Recurrence and Distant Metastasis Using Sample Size-Weighted Pooled Proportions.

Subgroup	Events/Total (n/N)	Estimate (%) *	95% CI ^†^
Local recurrence, per joint			
Knee	21/214	9.81	6.32–14.80
Shoulder	17/156	10.90	6.66–17.13
Hip	15/85	17.65	10.53–27.75
Exploratory Chi-square test, *p*-value	0.153		
Metastasis, per histologic subtype ^‡^			
Osteosarcoma	24/78	30.77	21.08–42.38
Soft tissue sarcoma	18/51	35.29	22.80–50.00
Chondrosarcoma	4/29	13.79	4.51–32.57
Ewing sarcoma	5/10	50.00	23.66–76.34
Exploratory Fisher’s exact test, *p*-value	0.088		

CI: Confidence interval. * Estimates were calculated using sample size-weighted pooled proportions. ^†^ Confidence intervals were calculated using the Wilson score method with continuity correction. ^‡^ For metastasis by histologic subtype, events/total (n/N) were calculated using available patient-level data; therefore, denominators may not correspond to the total sample size.

**Table 7:** Sensitivity analysis of sample size-weighted pooled local recurrence and distant metastasis after exclusion of studies including benign or metastatic lesions.

Outcome	Events/Total (n/N)	Estimate (%) *	95% CI ^†^
Local recurrence	41/308	13.31	9.83–17.74
Knee	17/125	13.60	8.35–21.16
Shoulder	16/132	12.12	7.30–19.22
Hip	8/51	15.69	7.48–29.14
Distant metastasis	93/258	36.05	30.25–42.27

CI: Confidence interval. * Estimates were calculated using sample size-weighted pooled proportions. ^†^ Confidence intervals were calculated using the Wilson score method with continuity correction.

**Table 8:** Contextual oncologic outcomes after intra-articular and extra-articular resection in comparative limb-salvage cohorts.

First Author	IAR Group			EAR Group		
	LR, n/N (%)	DM, n/N (%)	5-Year OS (%)	LR, n/N (%)	DM, n/N (%)	5-Year OS (%)
Knee						
Kawai et al. [34]	0/12 (0)	3/12 (25)	NR	0/28 (0)	2/28 (7)	NR
Kendall et al. [36]	0/9 (0)	0/9 (0)	NR	1/9 (11)	3/9 (33)	NR
Ieguchi et al. [19]	0/8 (0)	1/8 (13)	85	0/6 (0)	3/6 (50)	80
Shahid et al. [10]	4/21 (19)	9/21 (43)	67	12/42 (29)	18/42 (43)	60
Shoulder						
Meller et al. [33]	1/10 (10)	5/10 (50)	NR	1/10 (10)	1/10 (10)	NR
Böhler et al. [11]	0/32 (0)	NR	77	2/17 (12)	NR	59
Hip						
Fujiwara et al. [52] *	20/54 (37)	NR	NR	6/21 (29)	NR	NR

DM, distant metastasis; EAR, extra-articular resection; IAR, intra-articular resection; LR, local recurrence; NR, not reported; OS, overall survival. * Fujiwara et al. [52] was excluded from the present review because its extra-articular resection cohort overlapped with another included study [15] and is presented only as a contextual reference.

## Data Availability

The original contributions presented in this study are included in the article/Supplementary Materials. Further inquiries can be directed to the corresponding author.

## Supplementary Materials

The following supporting information can be downloaded at: https://www.mdpi.com/article/10.3390/cancers18142233/s1, Supplementary Table S1: PubMed and Embase Search Strategies and Retrieved Records; Supplementary Table S2: Quality Assessment of Included Cohort Studies Using the Newcastle–Ottawa Scale; Supplementary Table S3: Quality Assessment of Included Case Series Using the Joanna Briggs Institute Critical Appraisal Checklist; Supplementary Table S4: Preferred Reporting Items for Systematic Reviews and Meta-Analyses 2020 Checklist; Supplementary Table S5: Preferred Reporting Items for Systematic Reviews and Meta-Analyses 2020 for Abstracts Checklist.

## Abbreviations

The following abbreviations are used in this manuscript:

EAR	Extra-articular resection
LR	Local recurrence
DM	Distant metastasis
OS	Overall survival
CI	Confidence interval
LSS	Limb salvage surgery
IAR	Intra-articular resection
PRISMA	Preferred Reporting Items for Systematic Reviews and Meta-Analyses
LOE	Level of evidence
DF	Distal femur
PT	Proximal tibia
ST	Soft tissue
PF	Proximal femur
PH	Proximal humerus
ACJ	Acromioclavicular joint
NR	Not reported
OSA	Osteosarcoma
CS	Chondrosarcoma
UPS	Undifferentiated pleomorphic sarcoma
SS	Synovial sarcoma
ES	Ewing sarcoma
EM	Extensor mechanism
RT	Radiotherapy
CT	Chemotherapy
DFR	Distal femoral replacement
PTR	Proximal tibial replacement
APC	Allograft–prosthetic composite
T–L	Tikhoff–Linberg procedure
PHR	Proximal humeral replacement
LARS	Ligament Advanced Reinforcement System
EP	Endoprosthesis
PFR	Proximal femoral replacement
TFR	Total femoral replacement
W	Wide
M	Marginal
I	Intralesional
R0	Microscopically negative margin
R1	Microscopically positive margin
non-R0	Margin reported as not microscopically negative
JBI	Joanna Briggs Institute
NOS	Newcastle–Ottawa Scale

## References

[1] Ullah F., Altaf W., Khan D., Khan N.A., Akbar S., Muhammad Z. (2025). Comparative Outcomes of Limb Salvage Surgery Versus Amputation in Osteosarcoma: A Five-Year Follow-Up Study From a Tertiary Care Center. Cureus.

[2] Papakonstantinou E., Stamatopoulos A., Athanasiadis D.I., Kenanidis E., Potoupnis M., Haidich A.B., Tsiridis E. (2020). Limb-salvage surgery offers better five-year survival rate than amputation in patients with limb osteosarcoma treated with neoadjuvant chemotherapy. A systematic review and meta-analysis. J. Bone Oncol..

[3] Qi L., Ren X., Liu Z., Li S., Zhang W., Chen R., Chen C., Tu C., Li Z. (2020). Predictors and Survival of Patients with Osteosarcoma After Limb Salvage versus Amputation: A Population-Based Analysis with Propensity Score Matching. World J. Surg..

[4] Alamanda V.K., Crosby S.N., Archer K.R., Song Y., Schwartz H.S., Holt G.E. (2012). Amputation for extremity soft tissue sarcoma does not increase overall survival: A retrospective cohort study. Eur. J. Surg. Oncol..

[5] Enneking W.F., Spanier S.S., Goodman M.A. (2003). A system for the surgical staging of musculoskeletal sarcoma. Clin. Orthop. Relat. Res..

[6] Stauss R., Aigner A., Richter A., Suero E., Altemeier A., Savov P., Ettinger M., Omar M. (2023). The prognostic significance of surgical resection margins for local recurrence, distant metastasis, and overall survival in sarcoma. J. Surg. Oncol..

[7] Chen C.C., Wu Y.Y., Kao J.T., Chang C., Huang S.C., Shih H. (2024). Impact of resection margin on outcome in soft-tissue sarcomas of the extremities treated with limb-sparing surgery and postoperative radiotherapy. World J. Surg. Oncol..

[8] Li D., Xie L., Guo W., Tang X., Ji T., Yang R. (2018). Extra-articular resection is a limb-salvage option for sarcoma involving the hip joint. Int. Orthop..

[9] Zwolak P., Kühnel S.P., Fuchs B. (2011). Extraarticular knee resection for sarcomas with preservation of the extensor mechanism: Surgical technique and review of cases. Clin. Orthop. Relat. Res..

[10] Shahid M., Albergo N., Purvis T., Heron K., Gaston L., Carter S., Grimer R., Jeys L. (2017). Management of sarcomas possibly involving the knee joint when to perform extra-articular resection of the knee joint and is it safe?. Eur. J. Surg. Oncol..

[11] Böhler C., Brönimann S., Kaider A., Puchner S.E., Sigmund I.K., Windhager R., Funovics P.T. (2018). Surgical and Functional Outcome after Endoprosthetic Reconstruction in Patients with Osteosarcoma of the Humerus. Sci. Rep..

[12] Housset V., Anract P., Babinet A., Auberger G., Biau D. (2022). Proximal femur versus acetabular extra-articular resection of the hip joint for primary malignant bone tumors: A retrospective comparative review of 33 cases. World J. Surg. Oncol..

[13] Capanna R., van Horn J.R., Biagini R., Ruggieri P., Ferruzzi A., Campanacci M. (1990). The Tikhoff-Linberg procedure for bone tumors of the proximal humerus: The classical “extensive” technique versus a modified “transglenoid” resection. Arch. Orthop. Trauma Surg..

[14] Hardes J., Henrichs M.P., Gosheger G., Gebert C., Höll S., Dieckmann R., Hauschild G., Streitbürger A. (2013). Endoprosthetic replacement after extra-articular resection of bone and soft-tissue tumours around the knee. Bone Jt. J..

[15] Fujiwara T., Tsuda Y., Evans S., Stevenson J., Parry M., Jeys L., Abudu A. (2020). Extra-articular resection for bone sarcomas involving the hip joint. J. Surg. Oncol..

[16] Zoccali C., Papalia G.F., Cepparulo G., Baldi J., Sperati F., Amendola A., Salducca N., Gumina S. (2026). Extraarticular Knee Joint Resection: Indications, Results, and Complications in a Series of 30 Patients. Orthop. Surg..

[17] Anract P., Missenard G., Jeanrot C., Dubois V., Tomeno B. (2001). Knee reconstruction with prosthesis and muscle flap after total arthrectomy. Clin. Orthop. Relat. Res..

[18] Nottrott M., Streitbürger A., Gosheger G., Guder W., Hauschild G., Hardes J. (2019). Intra-articular soft-tissue sarcoma of the knee: Is extra-articular resection and tumor endoprosthetic reconstruction the solution? A retrospective report on eight cases. Orthop. Rev..

[19] Ieguchi M., Hoshi M., Aono M., Takada J., Ohebisu N., Kudawara I., Nakamura H. (2014). Knee reconstruction with endoprosthesis after extra-articular and intra-articular resection of osteosarcoma. Jpn. J. Clin. Oncol..

[20] Tsuda Y., Fujiwara T., Evans S., Kaneuchi Y., Abudu A. (2020). Extra-articular resection of shoulder joint for bone sarcomas: Oncologic and limb-salvage outcomes of 32 cases compared with shoulder disarticulation and forequarter amputation. J. Surg. Oncol..

[21] Schwab J.H., Healey J.H., Athanasian E.A. (2008). Wide en bloc extra-articular excision of the elbow for sarcoma with complex reconstruction. J. Bone Jt. Surg. Br..

[22] Sabourin M., Biau D., Babinet A., Dumaine V., Tomeno B., Anract P. (2009). Surgical management of pelvic primary bone tumors involving the sacroiliac joint. Orthop. Traumatol. Surg. Res..

[23] Xu M., Zheng K., Zhao J., Bai W., Yu X. (2019). En Bloc Resection and Pelvic Ring Reconstruction for Primary Malignant Bone Tumors Involving Sacroiliac Joint. Orthop. Surg..

[24] Angelini A., Mavrogenis A.F., Trovarelli G., Pala E., Arbelaez P., Casanova J., Berizzi A., Ruggieri P. (2017). Extra-articular shoulder resections: Outcomes of 54 patients. J. Shoulder Elb. Surg..

[25] Shekkeris A.S., Hanna S.A., Sewell M.D., Spiegelberg B.G.I., Aston W.J.S., Blunn G.W., Cannon S.R., Briggs T.W. (2009). Endoprosthetic reconstruction of the distal tibia and ankle joint after resection of primary bone tumours. J. Bone Jt. Surg. Br..

[26] Bennett G. (1947). Malignant neoplasms originating in synovial tissues (synoviomata); a study of thirty-two specimens registered at the Army Institute of Pathology during the war-time period, 1941–1945. J. Bone Jt. Surg..

[27] Karakousis C., Kontzoglou K., Driscoll D. (1998). Sarcomas near extremity joints in adults. J. Surg. Oncol..

[28] Khan I., Khan Z., Ahmad I., Khan A., Saeed M. (2022). Outcomes of the Combined Anteroposterior Approach For Forequarter Amputation in Shoulder Girdle Tumours. J. Ayub Med. Coll. Abbottabad.

[29] Zagra L. (2012). Abstracts from the 10Th Congress of the European Hip Society. HIP Int..

[30] Ruggieri P., Trovarelli G., Calabro T., Pala E., Angelini A., Maraldi M., Pieratelli G., Varela Osorio A.F., Piccioli A., Marcacci M. (2014). In-Depth Oral Presentations and Oral Communications. J. Orthop. Traumatol..

[31] Nakamura S., Kusuzaki K., Murata H., Takeshita H., Hirata M., Hashiguchi S., Hirasawa Y. (2001). Extra-articular wide tumor resection and limb reconstruction in malignant bone tumors at the proximal femur. Orthopedics.

[32] Chebib I., Rosenberg A.E., Fletcher C.D.M., Rosenthal D.I., Hornicek F.J., Nielsen G.P. (2016). Primary intra-articular sarcoma: A clinicopathological study of 15 cases. Histopathology.

[33] Meller I., Bickels J., Kollender Y., Ovadia D., Oren R., Mozes M. (1997). Malignant bone and soft tissue tumors of the shoulder girdle. A retrospective analysis of 30 operated cases. Acta Orthop. Scand..

[34] Kawai A., Muschler G.F., Lane J.M., Otis J.C., Healey J.H. (1998). Prosthetic knee replacement after resection of a malignant tumor of the distal part of the femur. Medium to long-term results. J. Bone Jt. Surg..

[35] Voggenreiter G., Assenmacher S., Schmit-Neuerburg K.P. (1999). Tikhoff-Linberg procedure for bone and soft tissue tumors of the shoulder girdle. Arch. Surg..

[36] Kendall S.J.H., Singer G.C., Briggs T.W.R., Cannon S.R. (2000). A functional analysis of massive knee replacement after extra-articular resections of primary bone tumors. J. Arthroplast..

[37] Wittig J.C., Bickels J., Kellar-Graney K.L., Kim F.H., Malawer M.M. (2002). Osteosarcoma of the proximal humerus: Long-term results with limb-sparing surgery. Clin. Orthop. Relat. Res..

[38] Capanna R., Scoccianti G., Campanacci D.A., Beltrami G., De Biase P. (2011). Surgical technique: Extraarticular knee resection with prosthesis-proximal tibia-extensor apparatus allograft for tumors invading the knee. Clin. Orthop. Relat. Res..

[39] Xie L., Tang X.D., Yang R.L., Guo W. (2014). Interscapulothoracic resection of tumours of shoulder with a note on reconstruction. Bone Jt. J..

[40] Samargandi R., Berhouet J., Nicolas Q., Le Nail L.R. (2025). The lighthouse technique for humeral suspension following a modified Tikhoff-Linberg procedure for the resection of bone and soft tissue tumors around the shoulder girdle: Clinical and functional outcomes. Eur. J. Orthop. Surg. Traumatol..

[41] Munn Z., Barker T., Moola S., Tufanaru C., Stern C., McArthur A., Stephenson M., Aromataris E. (2020). Methodological quality of case series studies: An introduction to the JBI critical appraisal tool. JBI Evid. Synth..

[42] Gualdi-Russo E., Zaccagni L. (2026). The Newcastle–Ottawa Scale for Assessing the Quality of Studies in Systematic Reviews. Publications.

[43] Stevenson M.G., Musters A.H., Geertzen J.H.B., van Leeuwen B.L., Hoekstra H.J., Been L.B. (2018). Amputations for extremity soft tissue sarcoma in an era of limb salvage treatment: Local control and survival. J. Surg. Oncol..

[44] Kirilova M., Klein A., Lindner L.H., Nachbichler S., Knösel T., Birkenmaier C., Baur-Melnyk A., Dürr H.R. (2021). Amputation for Extremity Sarcoma: Indications and Outcomes. Cancers.

[45] Rajasekaran R.B., Kurisunkal V., Stevenson J.D., Parry M.C., Morris G.V., Jeys L.M. (2024). A pictographic guide for decision making in surgery for pelvic bone sarcoma. J. Orthop..

[46] Coindre J.-M., Terrier P., Guillou L., Le Doussal V., Collin F., Ranchère D., Sastre X., Vilain M.-O., Bonichon F., Bui B.N. (2001). Predictive value of grade for metastasis development in the main histologic types of adult soft tissue sarcomas: A study of 1240 patients from the French Federation of Cancer Centers Sarcoma Group—PubMed. Cancer.

[47] Li R.H., Zhou Q., Li A.B., Zhang H.Z., Lin Z.Q. (2020). A nomogram to predict metastasis of soft tissue sarcoma of the extremities. Medicine.

[48] Zöllner S.K., Amatruda J.F., Bauer S., Collaud S., de Álava E., Dubois S.G., Hardes J., Hartmann W., Kovar H., Metzler M. (2021). Ewing Sarcoma-Diagnosis, Treatment, Clinical Challenges and Future Perspectives. J. Clin. Med..

[49] Khanna N., Pandey A., Bajpai J. (2017). Metastatic Ewing’s Sarcoma: Revisiting the “Evidence on the Fence”. Indian. J. Med. Paediatr. Oncol..

[50] Gazendam A., Popovic S., Parasu N., Ghert M. (2023). Chondrosarcoma: A Clinical Review. J. Clin. Med..

[51] Limaiem F., Davis D.D., Sticco K.L. (2023). Chondrosarcoma.

[52] Fujiwara T., Tsuda Y., Stevenson J., Parry M., Jeys L. (2021). Extra-articular resection of the hip joint for pelvic sarcomas: Are there any oncological and functional risks compared with intra-articular resection?. J. Bone Oncol..

